# Quantitative ultrasound assessment of lower limb muscle morphology in type 2 diabetes with and without peripheral neuropathy

**DOI:** 10.12688/f1000research.178108.1

**Published:** 2026-04-08

**Authors:** Sharath S, G Arun Maiya, Rajagopal Kadavigere, Prakashini K, Shivashankar Kaniyoor Nagri

**Affiliations:** 1 Department of Medical Imaging Technology, Manipal College of Health Professions, Manipal Academy of Higher Education, Manipal, Karnataka, 576104, India; 2 Centre for Podiatry & Diabetic Foot Care and Research, Department of Physiotherapy, Manipal College of Health Professions, Manipal Academy of Higher Education, Manipal, Karnataka, 576104, India; 3 Department of Radio diagnosis and Imaging, Kasturba Medical College, Manipal Academy of Higher Education, Manipal, Karnataka, 576104, India; 4 Department of Medicine, Kasturba Medical College, Manipal Academy of Higher Education, Manipal, Karnataka, 576104, India

**Keywords:** Quantitative ultrasound, Diabetic peripheral neuropathy, Muscle morphology, Lower limb muscles, Musculoskeletal ultrasound, Type 2 diabetes mellitus

## Abstract

**Background:**

Diabetic peripheral neuropathy (DPN) is associated with progressive skeletal muscle degeneration, particularly in distal lower limb muscles. Advanced imaging modalities such as CT and MRI can detect these changes; however, their routine clinical use is limited. This study aimed to evaluate the utility of quantitative musculoskeletal ultrasound for assessing lower limb muscle morphology in individuals with type 2 diabetes with and without DPN.

**Methods:**

In this cross-sectional study, 215 participants were included and categorized into three groups: T2DM with DPN (n = 129), T2DM without DPN (n = 43), and healthy controls (n = 43). DPN was assessed using the 10-g monofilament test and vibration perception threshold (VPT). Ultrasound measurements of the muscle thickness and cross-sectional area (CSA) were obtained for proximal and distal lower limb muscles using a standardized protocol. Group comparisons were performed using the Kruskal–Wallis test with DSCF post-hoc analysis. Associations were assessed using Spearman’s correlation.

**Results:**

Quantitative ultrasound revealed that participants with DPN demonstrated significantly reduced muscle thickness and CSA across most lower limb muscles compared with both T2DM without DPN and controls (p < 0.001), with the largest effect sizes observed in distal muscles, including tibialis anterior, extensor hallucis longus, extensor digitorum brevis, and abductor hallucis brevis. Ultrasound-derived muscle measurements showed significant correlations with neuropathy severity score and age.

**Conclusions:**

Quantitative musculoskeletal ultrasound can be used as a feasible and sensitive imaging modality for detecting diabetes-related muscle changes in the lower limb muscles enabling early detection and monitoring of neuromuscular involvement in type 2 diabetes.

**Abbreviations**
AHB: Abductor Hallucis Brevis
DPN: Diabetic Peripheral Neuropathy
EDB: Extensor Digitorum Brevis
EDL: Extensor Digitorum Longus
EHL: Extensor Hallucis Longus
RF: Rectus Femoris
SF: Subcutaneous fat
TA thickness: Tibialis Anterior Thickness
TA CSA: Tibialis Anterior Cross-Sectional Area
T2DM: Type 2 Diabetes Mellitus
TMT: Thigh Muscle Thickness
VI: Vastus Intermedius
WDPN: Without Diabetic Peripheral Neuropathy

## Introduction

Type 2 diabetes mellitus (T2DM) is the most prevalent metabolic disorder worldwide and is associated with a wide range of chronic complications affecting multiple organ systems. Among these, diabetic peripheral neuropathy (DPN) is one of the most common microvascular complications, affecting up to 50% of individuals with long-standing diabetes. DPN predominantly involves the distal and lower extremities and is characterized by sensory impairment, pain, distal lower limb muscle weakness, and progressive motor dysfunction, which together contribute to reduced mobility and impaired quality of life. ^1– 3^

Skeletal muscle plays a critical role in glucose metabolism and functional independence. In individuals with T2DM, chronic hyperglycaemia and insulin resistance contribute to progressive alterations in muscle structure and function. ^4– 6^ When peripheral neuropathy develops, denervation and motor unit loss further accelerate muscle atrophy, particularly in distal muscles of the lower limb. ^7, 8^ This progressive muscle degeneration increases the risk of gait instability, foot deformities, and ulceration, underscoring the importance of early and accurate assessment of muscle involvement in the diabetic population. ^7, 9^ Therefore, evaluating skeletal muscle mass and morphology in patients with DPN is of considerable clinical importance.

Accurate evaluation of muscle morphology has traditionally relied on imaging modalities, including computed tomography (CT), magnetic resonance imaging (MRI), and dual-energy X-ray absorptiometry (DEXA). Although these techniques provide precise estimates of muscle mass composition, their routine clinical use is limited by high cost, limited accessibility, radiation exposure, and logistical constraints. Consequently, there is a growing need for an imaging modality that is quantitative, reproducible, and feasible for use in outpatient and community-based settings. ^10– 12^ Musculoskeletal ultrasonography has emerged as a promising alternative for quantitative assessment of muscle morphology. Ultrasound enables the real-time evaluation of muscle thickness and cross-sectional area, it is free of ionizing radiation, and is portable and cost-effective. Previous studies have demonstrated strong correlations between ultrasound-derived muscle measurements and those obtained from CT and MRI and have shown potential for detecting early muscle changes in metabolic and neuromuscular conditions, making it well-suited for evaluating diabetes-related muscle degeneration. ^8, 13^ Despite increasing evidence supporting the use of ultrasound for muscle assessment, its application for systematic evaluation of lower limb muscle morphology in individuals with type 2 diabetes mellitus (T2DM), particularly in relation to peripheral neuropathy severity, remains limited. Therefore, the study aimed to quantitatively assess lower limb muscle thickness and cross-sectional area using musculoskeletal ultrasonography in individuals with and without diabetic peripheral neuropathy.

## Methods

### Ethics and registration

Ethical approval for the study was secured from Institutional Ethics Committee of Kasturba Medical College and Hospital, Manipal, India (Approval number: IEC496/2021, Date of approval October 22, 2021). The study was registered with the Clinical Trials Registry–India (CTRI; registration number: CTRI/2021/11/038116), with the registration date being November 18, 2021, and the participant enrolment began on November 22, 2021. The study was conducted in accordance with the principles outlined in the Declaration of Helsinki. Written informed consent was obtained from all participants. Participants provided consent for the use of anonymized data for research and publication purposes. No identifiable personal information is included in this manuscript.

### Study population

A group of 215 participants were recruited and categorized into three groups: individuals with type 2 diabetes mellitus (T2DM) with diabetic peripheral neuropathy (DPN) (n = 129), individuals with T2DM without DPN (n = 43), and healthy control participants (n = 43). Participants with type 2 diabetes mellitus (T2DM) were recruited from the institutional center for diabetic foot care, while healthy controls were recruited based on self-reported absence of diabetes and neuropathic symptoms.

The inclusion criteria consisted of participants diagnosed with T2DM, with and without peripheral neuropathy. Exclusion criteria were participants with type 1 diabetes, foot ulcers, fractures or implants in the lower limb, prior lower limb surgery or amputation, and a history of cerebrovascular accident. All participants provided written informed consent prior to participation.

### Assessment of Diabetic Peripheral Neuropathy (DPN)

Peripheral neuropathy was assessed in patients with type 2 diabetes mellitus (T2DM) using the 10-g Semmes-Weinstein monofilament test and vibration perception threshold (VPT) testing.

10-g monofilament test: The Semmes-Weinstein 10-g (5.07) monofilament (Diabetik Foot Care India, Chennai, India) was applied perpendicular to eight standardized sites on the plantar surface of the foot until the filament bent. Participants were asked to indicate their perception of the stimulus. Absence of sensation at one or more sites was considered indicative of peripheral neuropathy.

Vibration perception threshold (VPT): VPT was measured using a biothesiometer with participants in the supine position. The probe was applied to eight predefined anatomical sites, and participants were instructed to indicate when they first perceived the vibration. A vibration perception threshold of ≥15 V was considered indicative of diabetic peripheral neuropathy.

### Ultrasound examination

Musculoskeletal ultrasonography examination was performed using a Philips Epiq 5G ultrasound system with a linear array transducer (8–15 MHz). All examinations were performed with participants in the supine position.

Quantitative ultrasound measurements included muscle thickness and cross-sectional area (CSA) of selected proximal and distal lower limb muscles. Anatomical landmarks were identified using standardized protocols, and all measurements were obtained with minimal transducer pressure to avoid muscle compression. A generous coupling gel was applied to ensure optimal acoustic contact.

**Figure 1.**
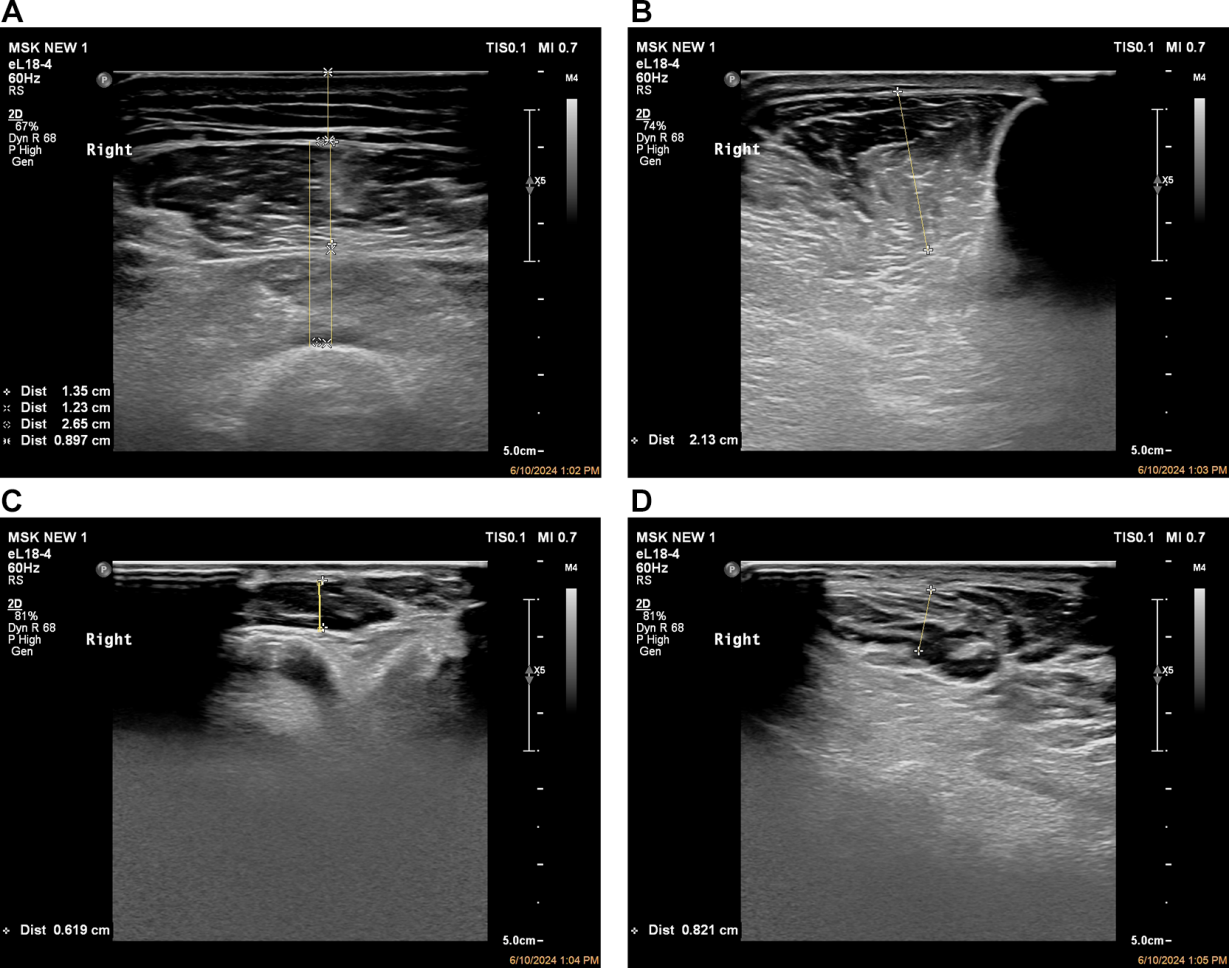
Quantitative musculoskeletal ultrasound assessment of lower limb muscles showing standardized probe position and muscle thickness measurement. **(A)** Measurement of the thickness of the anterior quadriceps muscle. **(B)** Ultrasound image of tibialis anterior demonstrating thickness and cross-sectional area in the transverse plane. **(C)** Thickness measurement of extensor digitorum brevis. **(D)** Thickness measurement of abductor hallucis brevis.

The following muscle groups were evaluated ( Figure 1):

Anterior thigh muscles: The midpoint between the greater trochanter and the superior border of the patella was identified. With the transducer placed transversely at this midpoint, measurements included subcutaneous fat thickness (SF), rectus femoris (RF), and vastus intermedius muscle (VI) thickness. Thigh muscle thickness (TMT) was calculated as the combined thickness of the rectus femoris and vastus intermedius muscles.

Ankle dorsiflexor muscles: The tibialis anterior (TA), extensor hallucis longus (EHL), and extensor digitorum longus (EDL) were assessed by positioning the transducer perpendicular to the long axis of the tibia at the point of maximum muscle belly thickness.

Intrinsic foot muscles: The extensor digitorum brevis (EDB) and abductor hallucis brevis (AHB) were evaluated with the transducer placed perpendicularly to the muscle belly. Measurements were obtained at maximal muscle thickness with minimal pressure.

All ultrasound measurements were performed by a single examiner trained in musculoskeletal ultrasonography to minimize inter-observer variability. Standardized scanning protocols and anatomical landmarks were used consistently across participants. To ensure measurement consistency, ultrasound system settings were kept constant throughout the study.

### Statistical analysis

Statistical analysis was performed using Jamovi software (Version 2.6.26). Data distribution for all variables was assessed using the Shapiro–Wilk test, which indicated non-normal distribution for multiple variables. Accordingly, non-parametric statistical methods were employed. Normally distributed variables are presented as mean ± SD; non-normally distributed variables are presented as median and interquartile range (IQR). Group comparisons among individuals with T2DM and DPN, those with T2DM without DPN, and healthy controls were performed using the Kruskal-Wallis H test. When significant, pairwise comparisons were conducted using the Dwass–Steel–Critchlow–Flinger post-hoc test. Effect sizes were reported using epsilon-squared (ε ^2^). A comparison between participants with and without DPN was performed using the Mann-Whitney U test, and effect sizes were expressed as rank-biserial correlations. Associations between ultrasound muscle measurements and demographic, neuropathic, and glycaemic variables were assessed using Spearman’s rank correlation coefficient. A p < 0.05 was considered statistically significant.

## Results

### Participant characteristics

A total of 215 subjects were included in the study: 129 individuals with T2DM and DPN, 43 individuals without DPN, and 43 healthy controls. The median age of the study population was 64 years (IQR: 56–70), and the median duration of diabetes among participants with T2DM was 14 years (IQR: 11–20). Baseline demographic, anthropometric, and clinical characteristics are summarized in Table 1. Shapiro–Wilk testing indicated non-normal distribution for most variables.

**Table 1.** Demographic and clinical characteristics of the study population.

Variable	Median (IQR)	Shapiro–Wilk W	p
Age (years)	64 (56–70)	0.941	< 0.001
Height (cm)	165 (158–170)	0.939	< 0.001
Weight (kg)	67.9 (59.0–73.2)	0.977	0.002
BMI (kg/m ^2^)	24.8 (22.6–27.1)	0.969	< 0.001
Duration of diabetes (years) *	14 (11–20)	0.912	< 0.001
FBS (mg/dL) *	132 (120–160)	0.916	< 0.001
PPBS (mg/dL) *	248 ± 80.3	0.978	0.015
HbA1c (%) *	7.5 (6.8–8.5)	0.935	< 0.001
VPT (V) *	25.0 (15.3–40.0)	0.940	< 0.001
Monofilament score (0–6) *	5.0 (4–6)	0.782	< 0.001

* Variables applicable only to participants with T2DM.

### Comparison of ultrasound measurements across groups

Quantitative ultrasound-derived muscle measurements were compared across the three groups using the Kruskal-Wallis H test, with effect sizes reported using epsilon-squared (ε ^2^). Significant differences were observed across most muscle parameters in Table 2.

**Table 2.** Comparison of ultrasound-derived lower limb muscle measurements among study groups.

Variable	χ ^2^ (df = 2)	p-value	ε ^2^	Significant Pairwise Differences (DSCF)
SF	5.40	0.067	0.025	NS between groups
RF	11.73	0.003	0.055	DPN < Control (p = 0.003)
VI	28.35	< 0.001	0.133	DPN < WDPN (p = 0.004); DPN < Control (p < 0.001)
TMT	18.10	< 0.001	0.085	DPN < Control (p < 0.001)
TA Thickness	33.48	< 0.001	0.157	DPN < WDPN (p = 0.002); DPN < Control (p < 0.001)
TA CSA	48.72	< 0.001	0.228	DPN < WDPN (p < 0.001); DPN < Control (p < 0.001)
EDL	52.05	< 0.001	0.243	DPN < Control (p < 0.001); WDPN < Control (p < 0.001)
EHL	62.93	< 0.001	0.294	DPN < WDPN (p < 0.001); DPN < Control (p < 0.001); WDPN < Control (p = 0.009)
EDB	54.90	< 0.001	0.257	DPN < WDPN (p < 0.001); DPN < Control (p < 0.001); WDPN < Control (p = 0.005)
AHB	58.80	< 0.001	0.275	DPN < WDPN (p = 0.007); DPN < Control (p < 0.001); WDPN < Control (p < 0.001)

Note: NS-Not significant.

Subcutaneous fat did not differ significantly among the groups (p = 0.067). In contrast, significant reductions in muscle thickness and cross-sectional area were observed in participants with DPN compared with both participants without DPN (WDPN) and healthy controls. Distal muscles demonstrated particularly large effect sizes, including tibialis anterior thickness (ε ^2^ = 0.157), tibialis anterior CSA (ε ^2^ = 0.028), extensor hallucis longus (ε ^2^ = 0.294), extensor digitorum brevis (ε ^2^ = 0.257), and abductor hallucis brevis (ε ^2^ = 0.257), indicating substantial differences between groups.

Post-hoc DSCF analysis revealed that participants with DPN had significantly lower muscle thickness and CSA than both participants without DPN and healthy controls for most distal muscles. Additionally, participants without DPN demonstrated intermediate muscle thickness values, with significant differences compared to healthy controls for select distal muscles, suggesting early muscle involvement even in the absence of clinically detectable neuropathy.

### Comparison between T2DM with and without peripheral neuropathy

Direct comparison between participants with DPN and those without DPN was performed using the Mann-Whitney U test ( Table 3). Participants with DPN exhibited significantly lower vastus intermedius thickness, thigh muscle thickness, tibialis anterior thickness, tibialis anterior CSA, extensor hallucis longus thickness, and extensor digitorum brevis thickness than participants without DPN (p < 0.05).

**Table 3.** Comparison of ultrasound muscle measurements between T2DM with and without DPN.

Variable	Mann–Whitney U	p-value	Rank-biserial correlation
SF	2302	0.096	0.170
RF	2261	0.070	0.185
VI	1879	0.002	0.323
TMT	2173	0.034	0.217
TA thickness	1814	< 0.001	0.346
TA CSA	1512	< 0.001	0.455
EDL	2298	0.093	0.171
EHL	1528	< 0.001	0.449
EDB	1692	< 0.001	0.390
AHB	1922	0.003	0.307

Effect size analysis demonstrated moderate to significant rank-biserial correlations for distal muscle measurements, particularly tibialis anterior CSA, extensor hallucis longus, and extensor digitorum brevis, indicating that distal muscle ultrasound parameters provide strong discrimination between diabetic individuals with and without peripheral neuropathy.

### Correlation between ultrasound measurements and clinical variables

Spearman’s correlation analysis demonstrated significant associations between ultrasound-derived measurements and demographic, neuropathic, and glycemic variables ( Table 4). Increasing age was negatively correlated with muscle thickness and CSA, particularly in distal muscles (ρ = −0.246 to −0.349, p < 0.001). Neuropathy severity, assessed using VPT and monofilament scores, showed significant negative correlations with tibialis anterior CSA, extensor hallucis longus, and intrinsic foot muscle thickness (p < 0.001). These findings indicate progressive muscle loss with increasing neuropathic severity.

**Table 4.** Correlation between ultrasound muscle measurements and clinical variables.

Variable pair	ρ (Spearman)	*p*-value
Age – Subcutaneous fat	−0.170	0.013
Age – TA thickness	−0.173	0.011
Age – TA CSA	−0.284	<0.001
Age – Distal muscles (EDL, EHL, EDB, AHB)	–-0.246 to −0.349	<0.001
Height – Vastus intermedius	0.141	0.040
Height – Thigh muscle thickness	0.143	0.037
Weight – Subcutaneous fat	0.213	0.002
BMI – Subcutaneous fat	0.263	<0.001
VPT – TA CSA	−0.299	<0.001
VPT – EHL	−0.304	<0.001
Monofilament – TA CSA	−0.201	0.009
HbA1c – FBS	0.701	<0.001
HbA1c – PPBS	0.757	<0.001
Vastus intermedius – Thigh muscle thickness	0.885	<0.001
Distal muscle inter-correlations (EDL, EHL, EDB, AHB)	0.30–0.40	<0.001

Across analyses, distal lower limb muscles consistently demonstrated larger effect sizes and stronger correlations with neuropathy severity compared to proximal muscles. Intrinsic foot muscles and ankle dorsiflexor exhibited excellent discriminative capacity between groups, highlighting their sensitivity to neuropathic changes and their potential utility as ultrasound imaging biomarkers for diabetic peripheral neuropathy.

## Discussion

The present study demonstrates that quantitative musculoskeletal ultrasonography can effectively detect morphological changes in the lower limb muscles of individuals with type 2 diabetes mellitus, particularly in the presence of diabetic peripheral neuropathy. Using standardized ultrasound measurements of muscle thickness and cross-sectional area, this study identified significant and progressive muscle loss across proximal and distal muscle groups, with the most significant degree of degeneration observed in the distal lower limb and intrinsic foot muscles. Importantly, these ultrasound-derived parameters showed strong associations with clinical measures of neuropathy severity, underscoring the potential of ultrasound as a quantitative imaging tool for neuromuscular assessment in the diabetic population. ^14– 17^

One of the key findings of this study is the higher sensitivity of ultrasound in detecting distal muscle involvement compared to proximal muscle changes. Distal muscles such as the tibialis anterior, extensor hallucis longus, extensor digitorum brevis, and abductor hallucis brevis demonstrated the largest effect sizes (ε ^2^ = 0.25–0.29) and strong correlations with VPT and monofilament scores. ^18, 19^ This pattern is consistent with the length-dependent nature of diabetic peripheral neuropathy, in which distal motor units are affected earlier and more severely due to axonal degeneration. From an imaging perspective, these findings suggest that distal muscle ultrasound measurements may serve as sensitive imaging biomarkers for early neuropathic involvement. ^7, 20, 21^

Previous imaging studies using MRI and CT have established the presence of muscle atrophy and fatty infiltration in individuals with diabetes and diabetic peripheral neuropathy. While these modalities provide a detailed assessment of muscle composition, their routine clinical use is limited by cost, availability, and logistical constraints. Musculoskeletal ultrasound offers several practical advantages, including portability, the absence of ionizing radiation, and real-time quantitative assessment. Prior studies have demonstrated strong correlations between ultrasound-derived muscle thickness and cross-sectional area, as measured by MRI and CT, supporting the validity of ultrasound as an imaging modality for muscle evaluation. The present study extends this evidence by demonstrating the applicability of ultrasound across multiple lower limb muscle groups and its association with neuropathy severity. ^22– 25^

A key observation in this study is that individuals with T2DM who do not have clinically detectable peripheral neuropathy also exhibit reduced muscle thickness in select distal muscles compared to healthy controls. This finding suggests that metabolic factors related to diabetes, such as insulin resistance and impaired protein synthesis, may contribute to early muscle alterations even before overt neuropathic symptoms develop. ^26– 28^ From an imaging standpoint, this highlights the potential role of ultrasound in the early detection of subclinical muscle changes in individuals with diabetes, which may have implications for preventive interventions. ^7^

The clinical imaging implications of these findings are significant. Quantitative musculoskeletal ultrasound may serve as a feasible imaging tool for screening and longitudinal monitoring of muscle health in individuals with diabetes. The ability to detect early distal muscle degeneration could aid in identifying patients at higher risk of functional decline, gait abnormalities, and diabetic foot complications. Furthermore, the non-invasive and cost-effective nature of ultrasound makes it suitable for repeated assessments in outpatient clinics and community healthcare settings, particularly in resource-limited environments. ^7, 9^

The observed association between ultrasound muscle measurements and neuropathy severity also supports the integration of muscle ultrasound with existing clinical assessment tools. While nerve conduction studies and sensory testing remain standard for diagnosing peripheral neuropathy, ultrasound provides complementary information by quantifying downstream muscular involvement. This integrated approach may enhance risk stratification and facilitate personalized management strategies aimed at preserving muscle function and mobility. ^7, 15^

This study has several limitations that should be acknowledged. First, the cross-sectional design limits the ability to evaluate the longitudinal progression of muscle degeneration or establish causal relationships between diabetic peripheral neuropathy and the changes in muscle. Second, functional outcomes such as muscle strength, gait, and balance parameters were not assessed, which may have provided additional insight into the clinical impact of the observed morphological changes. Future longitudinal and multicenter studies incorporating functional assessments and advanced imaging comparisons are required.

## Conclusions

This study demonstrates that quantitative musculoskeletal ultrasonography can reliably detect morphological changes in the lower limb muscles of individuals with type 2 diabetes mellitus, particularly in the presence of diabetic peripheral neuropathy. The distal lower limb and intrinsic foot muscles exhibited the most significant degree of atrophy and showed a strong association with neuropathy severity, highlighting their sensitivity to neuropathic involvement. These findings support the use of musculoskeletal ultrasound as a feasible, non-invasive imaging modality for the early detection and monitoring of diabetes-related neuromuscular changes in clinical practice.

## Ethics and consent

Study was approved by Institutional Ethics Committee of Kasturba Medical College and Hospital, Manipal, India [IEC496/2021] and the study was registered in Clinical Trial Registry India (CTRI) [CTRI/2021/11/038116]. The study was conducted in accordance with the principles outlined in the Declaration of Helsinki. Written informed consent was obtained from all participants. Participants provided consent for the use of anonymized data for research and publication purposes. No identifiable personal information is included in this manuscript.

## Supplementary material

Not applicable.

## Data Availability

The dataset supporting the findings of this study is openly available in Figshare: Quantitative Ultrasound Assessment of Lower Limb Muscle Morphology in Type 2 Diabetes with and Without Peripheral Neuropathy.” Figshare. https://doi.org/10.6084/m9.figshare.31291867. ^29^ The dataset includes:
• Quantitative lower limb muscle thickness measurements obtained from ultrasonography. All data have been fully de-identified in accordance with the Safe Harbor method, following HIPAA guidelines ( https://www.hhs.gov/hipaa/for-professionals/privacy/special-topics/de-identification/index.html#standard). The dataset is available under the terms of the Creative Commons Attribution 4.0 International license (CC-BY 4.0), which permits unrestricted use, distribution, and reproduction in any medium, provided the original work is properly cited. This study followed the STROBE (Strengthening the Reporting of Observational Studies in Epidemiology) guidelines for observational studies.
