# Supplementary material for: Quantitative ultrasound assessment of lower limb muscle morphology in type 2 diabetes with and without peripheral neuropathy
Source: F1000Res. 2026 Apr 8;15:488. [Version 1] doi: 10.12688/f1000research.178108.1 (PMC13103648; doi:10.12688/f1000research.178108.1)
Supplement: Peer review history

# Reviewer report: Reviewer response for version 1

**DOI:** 10.5256/f1000research.196453.r474338

**Published:** 2026-04-22

**Authors:** Rahul P Kotian

**Affiliations:** 1 RIMT University, Mandi Gobindgarh, Punjab, India; 2 Philips Middle East & Turkey & Africa, Dubai, United Arab Emirates

**Summary: -** This study evaluates the role of quantitative musculoskeletal ultrasound in assessing lower limb muscle morphology in individuals with type 2 diabetes mellitus with and without peripheral neuropathy. The authors found that muscle size is reduced especially in the lower leg and foot muscles, which are related to severity of nerve damage. The study suggests that ultrasound can be useful as a noninvasive tool for detecting and monitoring these changes.

**Strengths**

 1. Focus on clinically important topic related to muscle changes in diabetic neuropathy

 2. Result shows clearly the muscle changes in severity of peripheral neuropathy

**Minor Improvements**

 1. Reporting of mean and median can be standardized across variables.

 2. Minor language and formatting corrections can be made especially in reporting of p-values.

Is the work clearly and accurately presented and does it cite the current literature?

Yes

If applicable, is the statistical analysis and its interpretation appropriate?

Yes

Are all the source data underlying the results available to ensure full reproducibility?

Yes

Is the study design appropriate and is the work technically sound?

Yes

Are the conclusions drawn adequately supported by the results?

Yes

Are sufficient details of methods and analysis provided to allow replication by others?

Yes

Reviewer Expertise:

Medical imaging Technology, MRI, MSK Imaging, Computed Tomography

I confirm that I have read this submission and believe that I have an appropriate level of expertise to confirm that it is of an acceptable scientific standard.

# Reviewer report: Reviewer response for version 1

**DOI:** 10.5256/f1000research.196453.r474334

**Published:** 2026-04-20

**Authors:** Manna Debnath

**Affiliations:** 1 Radiography & Advance Imaging Technology, RSMAS, Royal Global University (Ringgold ID: 305831), Guwahati, Assam, India

**The author investigated the research entitled “Quantitative ultrasound assessment of lower limb muscle morphology in type 2 diabetes with and without peripheral neuropathy”.**

**Comments**

1. Introduction and methods are well written.
2. There is a discrepancy between the values reported in Table 2 and those described in the text on page 6. In Table 2, the ε² values for TA CSA and AHB are reported as 0.228 and 0.275, respectively, whereas in the text they are mentioned as 0.028 and 0.257. Kindly review and correct this inconsistency to ensure that the data presented in both the table and the text are accurate and consistent.
3.  The discussion and conclusions are well written.

Is the work clearly and accurately presented and does it cite the current literature?

Yes

If applicable, is the statistical analysis and its interpretation appropriate?

Yes

Are all the source data underlying the results available to ensure full reproducibility?

Yes

Is the study design appropriate and is the work technically sound?

Yes

Are the conclusions drawn adequately supported by the results?

Yes

Are sufficient details of methods and analysis provided to allow replication by others?

Yes

Reviewer Expertise:

Medical Imaging Technology

I confirm that I have read this submission and believe that I have an appropriate level of expertise to confirm that it is of an acceptable scientific standard.
